# Supplementary material for: Enhanced Cytotoxicity and Receptor Modulation by SMA-WIN 55,212-2 Micelles in Glioblastoma Cells
Source: Int J Mol Sci. 2025 May 9;26(10):4544. doi: 10.3390/ijms26104544 (PMC12111124; doi:10.3390/ijms26104544)
Supplement: Supplementary file 1 [file ijms-26-04544-s001.zip › ijms-3612893-supplementary.pdf]

Supplementary Table S1: Raw Cytotoxicity Data for Free WIN 55,212-2 and SMA-WIN in LN18 and A172 Glioblastoma Cell Lines

Cell Line	Treatment	Concentration (µM)	Cell Survival (% ± SEM)	IC50 (µM ± SEM)
LN18	Free WIN	5	95.0 ± 2.0	
		10	80.0 ± 1.8	
		20	52.0 ± 1.5	
		50	25.0 ± 1.2	20.97 ± 0.08
		100	10.0 ± 0.8	
LN18	SMA-WIN	5	90.0 ± 1.9	
		10	65.0 ± 1.6	
		20	30.0 ± 1.3	12.48 ± 0.11
		50	15.0 ± 0.9	
		100	5.0 ± 0.5	
A172	Free WIN	5	97.0 ± 2.1	
		10	85.0 ± 1.9	
		20	60.0 ± 1.7	
		50	28.0 ± 1.4	30.9 ± 0.12
		100	12.0 ± 0.9	
A172	SMA-WIN	5	92.0 ± 2.0	
		10	70.0 ± 1.8	
		20	35.0 ± 1.5	16.72 ± 0.09
		50	18.0 ± 1.0	
		100	6.0 ± 0.6	

Notes:

- Cell survival (%) was measured using the Sulforhodamine B (SRB) assay after 48 h treatment, relative to vehicle-treated controls (100%). Data represent mean ± SEM from three independent experiments.
- IC50 values were calculated using GraphPad Prism 7 via nonlinear regression.
